# Impact of Teachers’ Autonomy Support in Students’ Basic Psychological Needs, Intrinsic Motivation and Moderate-to-Vigorous Physical Activity

**DOI:** 10.3390/children10030489

**Published:** 2023-03-02

**Authors:** Alejandro Carriedo, José A. Cecchini, Antonio Méndez-Giménez, Deborah Sanabrias-Moreno, Carmen González

**Affiliations:** 1 Department of Education Sciences, Faculty of Teacher Training and Education, University of Oviedo, 33003 Oviedo, Spain; 2 Department of Didactics of Musical, Plastic and Body Expression, Faculty of Humanities and Educational Sciences, University of Jaén, 23071 Jaen, Spain

**Keywords:** accelerometers, physical activity, self-determination theory, physical education

## Abstract

The students’ active participation in physical education does not always guarantee the fulfilment of the international guidelines on moderate-to-vigorous physical activity (MVPA). The goal of the study was to examine the effects of the teacher autonomy support on the MVPA, basic psychological needs, and intrinsic motivation of primary education students. A three-phase, crossed research design was used in four groups of primary education (grades three, four, five, and six). Eighty-three students (40 boys; 43 girls) completed three physical education sessions with three levels of teacher autonomy support: low, intermediate, and high. They wore WGT3x accelerometers and answered a questionnaire. Results showed significant differences in MVPA, autonomy, competence, and intrinsic motivation (*p* < 0.001) between sessions one (controlling), two (intermediate-supportive autonomy), and three (full-supportive autonomy). In session three, the students’ MVPA increased to 73.70%. In conclusion, teacher’s autonomy-supportive environment can be modified to increase the students’ intrinsic motivation, basic physiological needs, and MVPA to reach the minimum international standards.

## 1. Introduction

Governmental guidelines indicate that school-aged children should be involved in at least 60 min of moderate-to-vigorous physical activity (MVPA) every day [1,2]. Unfortunately, only 20% of adolescents from across 105 countries meet this recommendation [3]. Schools are key settings for promoting physical activity (PA) [4], and physical education (PE) is the main subject. This area has the potential to provide students with regular occasions to be physically active, because up to 80% of school-aged children engage in PA exclusively in the school context [5]. Unfortunately, in many PE lessons, students do not reach sufficient MVPA levels to achieve health benefits [6]. A systematic review of PA levels concluded that students usually engage in MVPA for only 27–47% of lesson time [7]. In order to improve engagement in MVPA among students, factors within this context that are linked to habitual MVPA participation should be identified. The self-determination theory (SDT) [8] and the achievement goal theory (AGT [9]) are both theories of motivation that have been used to understand the factors of behavioral outcomes such as PA engagement.

SDT considers that the social environment operating within a specific context is a significant factor influencing the motivational processes and behavioral outcomes [7]. The social environment is created by the interpersonal behaviors of significant others acting within the context; in PE, teachers, and/or peers [7]. The teachers’ interpersonal behaviors are an outstanding factor influencing the teacher-created motivational climate [10]. Within SDT, three psychological needs have been identified. (a) Autonomy: Feelings that one is acting in a self-directed manner or the ability to feel in control of his/her behavior or destiny. (b) Competence: Beliefs that one can interact effectively with one’s environment, this need involves the feeling of being able to achieve desired outcomes. (c) Relatedness: Perceptions of being connected to significant others. This feeling occurs when someone is respected and cared for by others [11]. The teacher’s behaviors can affect the students’ motivation through the satisfaction of these three needs [12], which are considered as basic for children to improve their academic functioning and their personal development [13]. However, these needs require supportive class climates shaped by the teacher’s instructional style [14].

Deci et al. [15] believed that the teachers’ teaching style can be described along a continuum from total control or teacher-centered to autonomy-supportive or student-centered. In student-centered learning environments, teachers support the students’ personal improvement, fostering their autonomy [16]. They acknowledge the students’ interests, perspectives, thoughts, and feelings in goal setting and content selection; even allowing students to participate in these decisions [17]. Teachers explain the decisions and tasks selected [18]. They use inductive teaching styles where students play an active role [11], trying to remain close to their students answering questions, attending demands, or solving problems [14]. The goal is to help children self-regulate their learning [19]. In contrast, teachers that use directive teacher-led contexts do not want their students to actively participate in setting goals or selecting contents. Learning is externally regulated, and individual differences are not considered. The use of student-centered instructional strategies in PE is associated with greater student psychological development, well-being, psychological needs satisfaction, and learning [20].

Student motivation toward PE is positively associated with MVPA [21]. However, few studies have assessed the effects of interventions designed to motivate students to benefit from being involved in MVPA during PE [22]. Some studies in this field have examined the associations between motivation and PA levels in PE. For instance, Lonsdale et al. [12] found that students motivated by autonomous factors (e.g., intrinsic motivation) were 20% more active than students motivated by external factors (e.g., pressure from others). Similarly, the students’ autonomous motivation has been found to have a significant correlation to MVPA in PE lessons [23]. These findings indicate that PE teachers can play an important role in motivating school-age students to be actively involved in PE [13].

**Figure 1:**
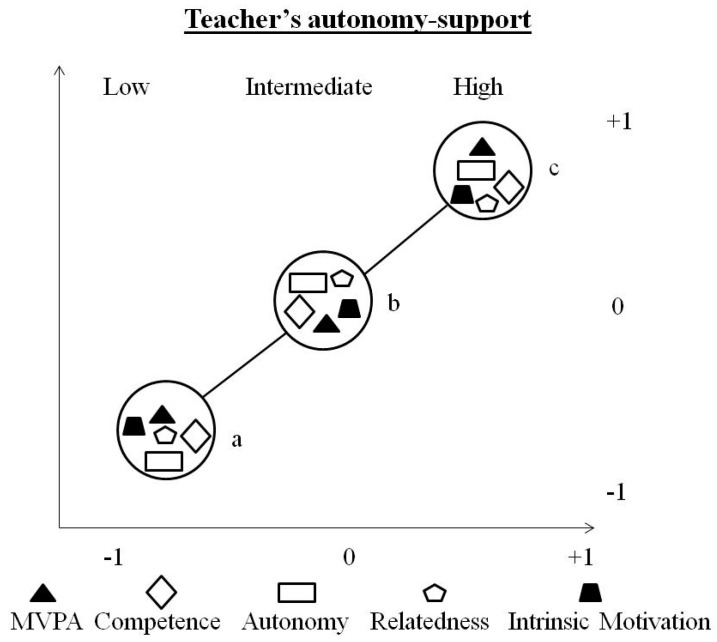
Graphical representation of the research hypothesis. Note. y-axis = levels of autonomy support; x-axis = basic psychological needs and intrinsic motivation.

Based on the aforementioned, and calls for field-based interventions to examine how one’s motivation for PE predicts objectively-assessed patterns of PA [24], the main goal of this study was to evaluate the effects of the teacher’s autonomy support on the primary education students’ MVPA, intrinsic motivation, and basic psychological needs. The initial hypothesis was that low, intermediate, and high levels of the teacher’s autonomous support will produce low, intermediate and high levels of the students’ basic psychological needs, intrinsic motivation, and MVPA, respectively (Figure 1).

## 2. Materials and Methods

### 2.1. Participants

In this study, 83 children (40 boys, 43 girls) participated with an age range of 8–12 years (*M* = 10.26; *SD* = 1.21). The sampling strategy was by convenience. They all belonged to four intact classes: 22 in grade three, 22 in grade four, 16 in grade five, and 23 in grade six of the same public school that is run on public funds located in northern Spain. Their experienced physical education teacher (more than ten years of teaching) also agreed to participate. Students attended two hours of physical education lessons per week (i.e., Monday and Wednesday).

### 2.2. Instruments

*MVPA*. This was assessed using Actigraph GT3X accelerometers. The GT3X is lightweight (27 g) and compact (3.8 cm × 3.7 cm × 1.8 cm). This activity monitor uses a solid state triaxial accelerometer to collect motion data on three axes and accurately and consistently measures time varying accelerations. These were initialized to measure PA in 10-s epochs. Verbal instructions were given by the researchers regarding how the accelerometer had to be worn and a demonstration was given. Participants were asked to wear the accelerometer on their right hip during all three PE sessions.

*Situational Basic Psychological Needs Scale*. This was based on the Basic Psychological Needs in Exercise Scale [25] and its validated Spanish version [26] to measure the situational competence, autonomy, and relatedness in primary education students. It contains 12 items (four per subscale) with the following prompt: “In this PE class…”; Autonomy (i.e., “I was able to take decisions”), Competence (i.e., “I felt capable of performing the tasks”), and Relatedness (i.e., “I got along with my classmates”). The Cronbach’s alpha was 0.96 for autonomy, 0.94 for competence, and 0.79 for relatedness.

*Situational Intrinsic Motivation Scale*. This was based on the Intrinsic Motivation subscale of the Motivation in Physical Education Questionnaire [27] to measure the situational intrinsic motivation. It contains four items (i.e., “I had fun”) with the following prompt: “In this PE class…”. The Cronbach’s alpha was 0.96.

In both questionnaires, the responses ranged from 1 (*completely disagree*) to 5 (*completely agree*).

### 2.3. Procedure

A three-phase (three sessions), crossed research design was used in four groups of primary education (grades three, four, five and six). Following the idea that the teachers’ teaching style can be described along a continuum that goes from total control to autonomy-supportive [18], three PE sessions were designed and implemented. In order to prevent a potential additive effect, there was one week between each session. Thus, the study was conducted in three consecutive weeks. The students’ autonomy support was progressively modified in the following elements: goals, content, teaching style, control, effort, pace, and grouping. All sessions were elaborated to fit three different levels of autonomy support: low (session one), intermediate (session two), and high (session three) using previous research works [28] and are outlined in Table 1. All PE classes (12 in total) were videotaped for analysis. These all lasted 40 min and were conducted by the same teacher. Each participant wore an accelerometer, and at the end of the class, they completed a questionnaire.

This project was implemented in three steps: first, the researchers obtained permission from the Ethics Committee, then the participants’ school administration permission was also obtained. Second, the teacher and all of the students’ parents signed their written informed consent. Third, after three regularly scheduled PE classes, all participants completed a questionnaire (they were also monitored via accelerometers).

Different studies have reported that PE teachers can be instructed to motivate their students more successfully [23,29]. Therefore, the procedure by Cheon et al. [29] for developing autonomy-supportive intervention programs was followed to train the participating teacher. It included the research team (with more than 10 years of theory, practice, and research on instruction) and the participating teacher, and it was divided into three parts. (1) Five-hour theoretical training including a reflective activity with two teaching scenarios, one scenario described highly autonomy-supportive teaching, and the other described a highly controlling teaching context. The teacher read both scenarios and answered questions about how well the scenarios described their own teaching. The students’ motivation, teacher styles, and ideas on “how to enact autonomy-supportive contexts” were also discussed. The teacher was asked to use this newly acquired knowledge in his PE classes and keep a structured diary answering the question: “How would you describe your teaching today: controlling or autonomy-supportive? Please explain”. (2) Five-hour workshop: This started with additional training on autonomy-supportive teaching and finished with a discussion group on the theory and the real practice experienced by the teacher in his classes; this was an opportunity to voice concerns, identify obstacles, and find possible solutions. Again, the teacher was asked to use the new information in his PE classes and preserve a structured diary of his efforts to enact an autonomy-supportive context. (3) Group discussion: Two five-hour sessions were held. The goal was to share and discuss ideas on how to develop autonomy-supportive environments in PE.

Fidelity of the implementation was assessed via trained observers/raters. Two raters, both professors at the University that conducted the study with more than 15 years of experience on PE teaching and SDT and were not related to the study, scored the teachers’ instructions and the students’ responses regarding the teacher’s autonomy-supportive or controlling style. They observed the videotaped sessions separately, not knowing to which group (low, intermediate, and high level of autonomy support) the lesson belonged. The rating sheet developed by Reeve et al. [30] was used and includes four instructional behaviors (relies on informational language, nurtures inner motivational resources, acknowledges and accepts expressions of negative affect, and offers explanatory rationales). In the current study, the ratings from the two observers were highly positively correlated in each behavior: 0.94, 0.97, 0.91, and 0.92, respectively. Therefore, the two ratings were averaged into a single score for each of the four autonomy-supportive instructional behaviors in the three sessions. Results obtained were as follows. (a) *Low versus intermediate autonomy support*: Nurtures inner motivational resources: *Ms*, 1.62 vs. 4.38; *t(14)* = −10.67, *p* < 0.001; relies on informational language: *Ms,* 1.13 vs. 3.88; *t(14)* = −8.59, *p* < 0.001; offers explanatory rationales: *Ms*, 1.37 vs. 4.50; *t(14)* = −9.65, *p* < 0.001; and acknowledges and accepts expressions of negative affect: *Ms*, 1.50 vs. 4.25; *t(14)* = −7.51, *p* < 0.001, *d*= 1.17. (b) *Intermediate versus high autonomy support*: Nurtures inner motivational resources: *Ms*, 4.38 vs. 6.50; *t(14)* = −8.08, *p* < 0.001; relies on informational language: *Ms*, 3.88 vs. 6.88; *t(14)* = −9.36, *p* < 0.001; offers explanatory rationales: *Ms,* 1.37 vs. 4.50; *t(14)* = −9.65, *p* < 0.001; and acknowledges and accepts expressions of negative affect: *Ms,* 4.25 vs. 6.50; *t(14)* = −6.15, *p* < 0.001. The significant differences observed between intermediate and high makes it unnecessary to show the results between low and high autonomy support. Results showed that each lesson was conducted according to the teacher autonomy support expected.

### 2.4. Data Analysis

Data were analyzed using SPSS 22.0 (IBM). First, exploratory analyses, the Kolmogorov–Smirnov test, were run to determine the parametric properties of the data. Repeated measures *t*-tests were used for the parametric data and the Wilcoxon rank test for the non-parametric data. To assess the group differences, the independent *t*-test was used for the parametric analyses while the Mann–Whitney *U* test was used for non-parametric analyses. Finally, the effect size (ƒ) was calculated and included with its corresponding significant outcomes (small = 0.20, moderate = 0.50, large = 0.80 [31]).

## 3. Results

**Figure 2:**
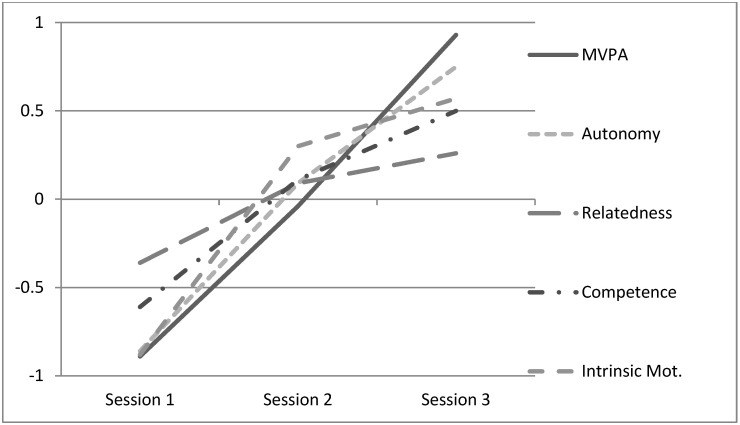
MVPA, basic psychological needs, and intrinsic motivation in sessions one, two, and three.

Table 2 shows the descriptive analyses (means and standard deviations) of all variables assessed in sessions one, two, and three. Significant differences were obtained in all variables (MVPA, autonomy, competence, and intrinsic motivation), except for the relatedness between sessions two and three. MVPA percentage increased from 38.65% in session one to 54.92% in session two, and 73.70% in session three. The effect size can be considered as large (>0.80) between sessions one, two, and three in all variables, except for relatedness. The effect size between sessions two and three was inexistent in the variable relatedness, small in intrinsic motivation, moderate in competence and autonomy, and large in MVPA. Figure 2 represents the different scores among variables in the three tested sessions. Table 3 shows the PA levels (sedentary, light, moderate, vigorous) in the three testing sessions. A decrease in sedentary and light and an increase in moderate and vigorous PA between sessions one, two, and three was observed. Differences were statistically significant in all cases (*p* < 0.001).

## 4. Discussion

The main goal of the study was to assess the effects of the teacher’s autonomy support on the primary education students’ basic psychological needs, intrinsic motivation, and MVPA. The initial hypothesis was that low, intermediate, and high levels of teacher’s autonomous support will produce low, intermediate, and high levels of the students’ basic psychological needs, intrinsic motivation, and MVPA, respectively. Our results support the initial hypothesis, except in relatedness between the intermediate (session 2) and high levels of autonomy support (session 3).

Based on the idea that the teachers’ teaching style can be described along a continuum that goes from total control to autonomy-supportive [18], and that PE teachers can be instructed to motivate their students more successfully [29], three PE sessions for primary education students in grades 3, 4, 5, and 6 were designed and implemented, progressively modifying the students’ autonomy support in the following elements: goals, content, teaching style, control, effort, pace, and grouping. When the teacher set the goals and selected the content, used direct instruction, and regulated the students’ learning, forcing students to repeat the tasks with no individualization or cooperation among students and focused on the outcomes, thus creating a context of low level autonomy support, the students’ basic psychological needs (particularly autonomy), intrinsic motivation, and MVPA levels were low. Therefore, the controlling teaching style produced MVPA scores below 40%, not reaching the minimum recommendations. These results are also in line with those from Fairclough and Stratton [7], who found that secondary and primary education students reached the MVPA levels in 27–47% of the PE class. These findings show the detrimental effects of controlling teaching for the students’ MVPA, basic psychological needs, and intrinsic motivation. Teachers and educators should be aware of these risks.

In session two, the teacher was more autonomy supportive and significant changes were obtained in all variables. Moreover, the effect size was large in all of them, from 0.62 in relatedness to 3.26 in MVPA and was larger between sessions one (low autonomy support) and two (intermediate autonomy-support) than between sessions two and three (high autonomy support). The teacher still set the goals and selected the content, but he explained the reasons behind those decisions, providing extra information. The most important changes occurred in the teaching style, which shifted from direct instruction to problem solving, and in the control, where students were allowed to self-regulate (the teacher intervened correcting mistakes). The MVPA levels increased, meeting the previously mentioned international recommendations [1,2]. This finding means that if teachers modify their autonomy support in their PE classes at an intermediate level, acceptable levels of MVPA can be reached.

Finally, in session three, when the teacher allowed the students to participate in goal setting and content selection, he built his teaching style on problem solving practices, so the students had to solve challenges through individualized self-regulatory learning within a cooperative structure, and the effort and improvement were recognized, and the students’ basic psychological needs, intrinsic motivation, and MVPA continued improving. Previous research has linked the teachers’ teaching strategies, the students’ basic psychological needs, and MVPA [20]. In the present study, when students felt that there was higher teacher autonomy support, they also showed higher levels of autonomy. Our results are in line with those from previous research, which found that teachers who use instructional strategies to support student autonomy produced positive effects on their autonomy satisfaction [32,33]. In this same trend, higher teacher autonomous support produced higher perceived student competence, which was also observed in previous research [34]. In the PE classes, Haerens et al. [35] found that the teacher’ s autonomy support increased the students’ basic psychological needs while controlling contexts decreased them. Similarly, intrinsic motivation also increased as the teacher’s autonomous support increased. Strategies used to support the students’ autonomy are considered as a key social factor in the development of motivation in class contexts [36] or enjoyment in PE classes [37]. Finally, the MVPA levels significantly increased as the teacher’s autonomous support increased: from 38.65% in session one (low) to 73.7% in session three (high). These results are consistent with those found in previous studies where the students’ PA was measured via accelerometers [22,23].

Results from the present study showed that PE teachers can be autonomy-supportive at specific times (i.e., tasks, sessions) and might produce significant improvements in the students’ intrinsic motivation, basic psychological needs, and MVPA. Previous research works have found that the more teachers used autonomy support in their PE classes, the more engaged were their students [14]. Our results showed that even when teachers were autonomy-supportive at an intermediate level, they might produce significant effects in their students. Despite the positive effects linked to the students’ autonomy support, the tendency among PE teachers was toward the use of more controlling styles [38], which might negatively influence the students’ basic psychological needs, intrinsic motivation levels, and MVPA [35]. An autonomy-supportive class climate in PE has been found to produce positive outcomes in the students [36]. In contrast, exposing the students to more controlling class contexts might trigger negative consequences [39]. Teachers and teacher educators should be aware of this to benefit their students.

The present study had several limitations. First, the number of participants could be considered as limited. The procedure followed was based on a within subject design, which allows researchers to examine differences between conditions with fewer participants than the between subject design sample sizes [40]. In this study, participants of each group underwent all conditions (low, intermediate, and high level of autonomy). Similar studies in this field have drawn conclusions from smaller samples sizes [41]. Thus, although the sample size could be considered enough in this study when searching for inferences and the *p* value observed (*p* < 0.001) suggested that there was a low risk of committing type 1 errors, similar studies should try to find significant results with higher sample sizes in order to confirm these results. Second, the short-term nature of the intervention, the effect of time on students, and the sampling strategy could also be considered as potential limitations. Likewise, the PA habits of the participants could have been collected in order to have a better understanding of the results of this study. Finally, it should be acknowledged that the design of this study does not guarantee a lack of additive effect. However, the three-phase, crossed research design conducted in four different groups is a strength of this study. In conclusion, PE teachers can modify their teaching style to make it more autonomy-supportive and increase the primary education students’ MVPA (to reach international recommendations), basic psychological needs, and intrinsic motivation. Future research should analyze these relationships with larger samples and students from other contexts. Moreover, it could be interesting to examine how other variables could affect these results such as the physical activity habits of students, their commitment in participating in sporting activities, or with other PE teachers.

## Figures and Tables

**Table 1:** Teacher’s autonomy support in session one, two, and three.

Low	Intermediate	High
Goals		
These were set by the teacher. No explanations were given to the students	These were set by the teacher, but the selection was fully explained to the students	Students participated in goal setting
Content		
It was selected by the teacher. Students were not asked	It was selected by the teacher, but the students were informed of the reasons behind the selection	The students’ perspectives, feelings, thoughts, and ideas were fully considered
Teaching Style		
Direct instruction was used. All students were asked to perform identically	Problem solving was used. Students were asked to find different solutions to the problem set by the teacher	Problem solving was used, but the students were asked to set different problems and find creative solutions
Control		
External, by the teacher; based on the repetition of tasks	Students were allowed to self-regulate, but the teacher intervened, detecting and correcting mistakes	Students were supported to self-regulate their learning, to understand difficulties and errors autonomously
Effort		
Outcomes or end-results were recognized (product)	Effort and participation were recognized (process)	Effort and improvement, based on the students’ ability to self-regulate their leaning, were recognized
Pace		
This was the same for all students (their starting point was not considered)	This was the same for all students, but at the end of every task, they were informed of their improvements	It was not the same for every student. Each was allowed to self-regulate their practice time
Grouping		
No groups were selected	Groups were selected by the teacher	Groups were selected by the students

**Table 2:** Means, standard deviations, and differences in MVPA, basic psychological needs, and intrinsic motivation in sessions one (low autonomy-supportive teaching), two (intermediate autonomy-supportive teaching), and three (high autonomy-supportive teaching).

	Session 1 *M* (*SD*)	Session 2 *M* (*SD*)	Session 3 *M* (*SD*)	Difference S1–S2	ƒ	Difference S2–S3	ƒ	Difference S1–S3	ƒ
MVPA	11.38 (2.89)	21.97 (3.57)	29.48 (4.55)	*T_79_* = −22.39 ***^a^	3.26	*T_79_* = −13.31 ***^a^	0.67	*T_79_* = −29.48 ***^a^	4.71
Autonomy	1.75 (0.97)	3.56 (1.44)	4.60 (0.89)	Z_79_ = −6.87 ***^b^	1.62	Z_79_ = −5.40 ***^b^	0.87	Z_79_ = −7.67 ***^b^	3.25
Relatedness	4.07 (0.99)	4.57 (0.61)	4.68 (0.64)	Z_79_ = −4.26 ***^b^	.61	Z_79_ = −1.65 ^b^	0.09	Z_79_ = −4.80 ***^b^	0.73
Competence	3.19 (1.41)	4.36 (0.86)	4.76 (0.49)	Z_79_ = −5.94 ***^b^	1.00	Z_79_ = −4.15 ***^b^	0.57	Z_79_ = −6.97 ***^b^	1.45
Intrinsic Motivation	2.27 (1.35)	4.42 (0.87)	4.78 (0.62)	Z_79_ = −7.31 ***^b^	1.89	Z_79_ = −3.34 ***^b^	0.23	Z_79_ = −7.52 ***^b^	2.39

*Note*. a = *t*-test; b = Wilcoxon rank test; ƒ = effect size. *** *p* < 0.001

**Table 3:** Means, standard deviations, and percentages of sedentary, light, moderate, and vigorous physical activity in sessions one (low autonomy-supportive teaching), two (intermediate autonomy-supportive teaching), and three (high autonomy-supportive teaching).

	Session 1		Session 2		Session 3		Diff	Diff	Diff
	*M* (*SD*)	%	*M* (*SD*)	%	*M* (*SD*)	%	S1–S2	S2–S3	S1–S3
Sedentary	19.41 (6.29)	48.50	13.76 (3.39)	34.44	7.03 (3.39)	17.58	Z_79_ = 6.13 ***^b^	Z_79_ = 7.45 ***^b^	Z_79_ = 7.70 ***^b^
Light	5.13 (1.48)	12.82	4.26 (1.19)	10.64	3.49 (1.62)	8.72	*T_79_* = 4.30 ***^a^	*T_79_* = 3.65 ***^a^	*T_79_* = 3.84 ***^a^
Moderate	12.92 (4.52)	32.31	15.88 (2.72)	39.70	21.48 (3.78)	53.71	Z_79_ = −5.11 ***^b^	Z_79_ = −7.63 ***^b^	Z_79_ = −7.63 ***^b^
Vigorous	2.54 (3.12)	6.37	6.10 (2.23)	15.22	8.00 (4.34)	19.99	*T_79_* = −8.31 ***^a^	*T_79_* = −4.42 ***^a^	*T_79_* = −10.06 ***^a^

*Note*. a = *t*-test; b = Wilcoxon rank test. *** *p* < 0.001.

## Data Availability

Data are available for research. Any further inquiries can be directed to the authors.
